# Lupus of the Upper Air Passages

**Published:** 1908-03-07

**Authors:** 


### Laryngology and Rhinology.

### LUPUS OF THE UPPER AIR PASSAGES.

### Ætiology.

Lupus of the nasal passages, pharynx, or larynx is not in reality an altogether rare affection, but it produces so little discomfort in the slight and early forms that it is then frequently overlooked. Though occasionally met with in the absence of cutaneous lesions, it is nearly always found in association with lupus vulgaris of the skin, and it therefore appears most frequently in females between the ages of twelve and twenty. Indeed, if a large number of cases of cutaneous lupus be carefully examined, probably in 20 per cent. deposits will also be found in some part of the upper air-passages. It is most often found in the nose, and becomes rarer as we descend the respiratory tract, being more often seen on the palate and pharynx than in the larynx. Indeed, the favourite sites of its occurrence afford strong presumptive evidence that it owes its origin to direct infection of the nasal mucous membrane, and that it spreads thence downwards. Thus the common site for its inception in the nose is a point on the septum just within the nostril and well within reach of the finger in the same position that erosions, largely due to picking, are often found; the front end of the inferior turbinal is affected the next in frequency. In the pharynx the posterior surface and edges of the soft palate are most usually attacked, precisely the parts one would expect to find diseased if the infecting agent is carried by discharges from the nose; so also in the larynx it is the tip and edges of the epiglottis which first show signs of the disease.

As to the actual causal agent, there is little doubt that it is the bacillus tuberculosis, which has been found in small numbers in the tissues, and whose presence can be more readily proved by inoculation experiments. Further, the lesions react to injections of tuberculin, and many cases of lupus eventually die of phthisis. But it is nevertheless something of a puzzle that on the skin, pharynx and larynx the clinical appearance of lupus is absolutely distinct from that of tuberculosis. It may be that the organism is a variety of the tubercle bacillus.

Escat* has propounded an ingenious theory to the effect that the tubercle bacilli first gain a lodgment on the nasal mucous membrane, where the unfavourable surroundings modify their virulence, and that all cases of lupus therefore begin within the nose. The position and order of invasion of the various parts of the upper air-passages mentioned above would appear to bear out this hypothesis. It is certainly remarkable that lupus and tuberculosis are so distinct clinically in other parts of the upper respiratory tract, but in the nose are practically indistinguishable. Acute tuberculous ulceration, such as is well known in the larynx and pharynx, occurs rarely, if at all, in the nose. Escat, indeed, denies the occurrence of true tuberculosis in the nasal passages, and classes all such cases as lupus. On the other hand, the disease can by no means always be detected within the nose in cases of cutaneous lupus; but it may be said that in such a complex region a small lesion or a healed ulcer might easily escape detection.

* "Annales des Maladies de l'Oreille," etc. Vol. xxxi. No. 10. 1905.

### Subjective Symptoms.

The symptoms resulting from this affection are remarkably slight and inconspicuous, and for this reason many cases remain undetected if the skin is free from the disease or unless the upper respiratory tract of patients suffering from lupus is systematically examined. Pain is very slight or altogether absent, even when there is extensive ulceration. Hoarseness is the principal symptom when the larynx is attacked, but this organ is usually only affected late in the case, and even then the vocal cords escape for some time. In the stage of contraction dyspnœa may result, and stenosis of the pharynx may cause obstruction to swallowing.

### Objective Symptoms.

On the mucous membranes, as on the skin, the disease first shows itself as a dusky red nodular infiltration; the nodules later become more translucent and brownish in colour, producing the well-known "apple-jelly" appearance. These are rendered more apparent on the skin by pressing a watch-glass on to the surface, so as to make the part bloodless, and on the mucous membrane the same result can be obtained by the application of adrenalin, which shows up the dusky infiltration very clearly against the pallor of the healthy mucosa. Later the nodules break down and ulceration commences; the ulcers are superficial with smooth, dry indolent base, and well-defined serpiginous margin; they extend very slowly in places and cicatrise in others, forming a dense scar-tissue, which contracts and, in severe cases, produces great deformity. The whole process is very insidious and chronic, and the only danger to life lies in secondary stenosis and in a tendency to phthisis; many subjects of lupus die eventually of the latter disease.

In the nose lupus generally begins on the front and lower part of the septum; ulceration occurs, and perforation of the triangular cartilage is common, but the bony septum is not involved. The disease also attacks the inner aspect of the ala nasi, and often produces a circular perforation in this region, and stenosis of the nostril frequently occurs. On the fauces the lesions are usually seen on the uvula and edges of the soft palate; the latter may be much thickened and distorted or may be completely eaten away. Perforation in the centre of the palate does not occur. In bad cases the pharyngeal wall is also affected, and adhesion of palate and pharynx may be produced, giving an appearance very similar to that which is caused by syphilitic ulceration. In the larynx the tip and edges of the epiglottis are first attacked, and the part is gradually ulcerated away; sometimes the nodules are absorbed without ulceration, and a characteristic pitted appearance results. The disease slowly spreads along the arytenoepiglottidean folds, but the cords generally remain immune for a long time.

Diagnosis.

This is in general easy, especially as the characteristic lesions are usually present on the skin. In the larynx and fauces the disease differs from tuberculosis in almost every particular—situation, painlessness, nodular deposit, and formation of scar-tissue. In the late stage of cicatricial contraction the affection may be indistinguishable from syphilis by the appearance alone: perforation of the bony septum or of the hard palate does not occur in lupus; syphilitic ulcers are deeper and the granulations larger and more exuberant. The disease in the nose may easily be overlooked or mistaken for simple erosion; the use of adrenalin is a great aid to the detection of the nodules.

Treatment.

Open-air treatment at a sanatorium has a most beneficial effect. Arsenic, given in gradually increasing doses, may almost be considered as a specific. Local treatment is often of great value, but it will frequently be found that less need be done than might have been expected after a course of general treatment has been applied; this should always be done first, and the remaining intractable lesions should then be surgically treated. It is useless to scrape the skin-lesions of facial lupus without paying due attention to the intra-nasal disease at the same time. Local treatment consists of the removal of affected parts, such as the epiglottis or uvula, thorough scraping away of infiltration and ulceration, and subsequent applications of caustics, of which lactic acid may be recommended. This can usually be done under local anæsthesia, and, to make sure of treating the entire affected area, adrenalin should also be applied. If the disease be extensive in the nose its removal can sometimes be more thoroughly completed under a general anæsthetic. Recurrence is common, and all cases should be afterwards examined at regular intervals. Localised recurrences can be treated with the best results by the application of the galvano-cautery to the nodules.

